# News and Coming Events

**Published:** 1907-09-28

**Authors:** 


### NEWS AND COMING EVENTS.

The Royal Dental Hospital, Leicester Square, has received the sum of £250, less legacy duty, from the executors of the late Mr. John Lawrence Toole.

The High Court of Germany has decided that it is illegal for the physicians of any locality to band together and establish a definite code of fees for professional services.

The inaugural public lecture at King's College will be delivered at 4 p.m. on Wednesday, October 2, by Professor Peter Thompson, M.D., who has taken as his subject "The Study of Embryology."

A concert in the series of twenty Odeon Concerts in aid of the Lord Mayor's Cripples' Fund, and under the Lord Mayor's patronage, was given at Hammersmith Town Hall on Friday, September 20. Sir John Kirk, the Secretary of the Ragged School Union, presided, and opened the proceedings with a short speech, pleading very earnestly for the cripples and the cause which the Odeon Company has taken up. The record specially made for these concerts by the Lord Mayor was recited, and could be heard clearly all over the hall. An interesting programme has been arranged for future concerts.

St. John's Hospital for Diseases of the Skin, Leicester Square, W.C.—The Chesterfield Lectures, founded in 1895 in connection with a silver medal presented by the Earl of Chesterfield to promote the study of dermatology (and which is open for competition to those who have attended three-fourths of the lectures), are free to medical practitioners on presenting their cards and to medical students who desire to attend regularly, and will be resumed at 49 Leicester Square on Thursday evening, October 3, at 6 p.m. The Chesterfield lecturer, Dr. Morgan Dockrell, will give his opening lecture on "The Present Position of Dermatology." After each lecture demonstrations will be given on special cases, followed by clinical instruction up to eight o'clock on patients presenting themselves in the outpatient department. The lectures are essentially practical, and deal fully with diagnosis and treatment, being illustrated by large diagrams, clinical and microscopical, specially prepared for each lecture.

The Lord Mayor of London laid the foundation-stone of a new wing of the Buchanan Hospital, St. Leonards-on-Sea, on Tuesday, September 24. This hospital was founded in 1881, mainly by the beneficence of the late Miss Elizabeth Merrlees, and since then has been considerably enlarged and extended. It consists of 19 beds in the general wards and three private wards, with a distinct building for out-patients. Last year 246 in-patients were treated, and there were 8,933 out-patients' attendances. For some time past the need of a children's ward has become very apparent, and in March last the late Mrs. Thomas Mason gave £3,000 for the erection of a children's wing. Plans of a handsome building were prepared by Mr. H. Ward, A.R.I.B.A., of Hastings. As, by the donor's wish, the bulk of the money has to be expended on the building, the hospital is in urgent need of funds in order that the considerable increase of expense involved in this large addition may be met. The new wing will consist of a large ward, to contain from 12 to 18 cots, with a verandah (leading out on to a playground), board-room, secretary's office, and an isolation ward, with kitchen, bath-rooms, etc., complete.

The medical session of King's College opens on October 1 next, when the annual distribution of prizes to successful students will take place. The opening address will be delivered by Dr. W. H. Allchin, M.D., F.R.C.P., Consulting Physician to the Westminster Hospital, at 4 p.m. The museums and laboratories will be open to visitors.

The surgical dispensary for fisherfolk at Point Law, Aberdeen, was closed early in the present month after a long and unusually busy season. The number of dressings done exceeded 1,100, while the number of patients treated was 345. The dispensary fulfils a want, and the good work that it does yearly is thoroughly appreciated by the fisherfolk.

### ST. BARTHOLOMEW'S HOSPITAL AND MEDICAL SCHOOL.

During the past year the following changes have taken place in the teaching staff :—

Dr. Christopher Addison, who was formerly Dean of Charing Cross Medical School and Professor of Anatomy at the University College, Sheffield, has been appointed Lecturer and Senior Demonstrator of Anatomy.

Mr. W. D. Harmer has resigned the Assistant Surgeoncy, and has been appointed Surgeon in charge of the Department for Diseases of the Throat and Nose. Mr. F. A. Rose has been appointed Assistant Surgeon for Diseases of the Throat and Nose. Mr. G. E. Gask has been appointed Assistant Surgeon and Teacher of Clinical Surgery.

Dr. W. S. A. Griffith has been elected Physician Accoucheur with charge of out-patients, and will give part of the clinical lectures on Diseases of Women. Dr. H. Williamson has been elected Assistant Physician Accoucheur and Clinical Lecturer in Midwifery.

Dr. J. A. Willett has been appointed Demonstrator of Midwifery. Mr. C. E. West has been elected Assistant Aural Surgeon. Mr. C. Gordon Watson has been appointed Surgical Registrar, and has resigned the Demonstratorship of Anatomy.

Mr. L. B. Rawling has been appointed Demonstrator of Operative Surgery. Mr. R. C. Ackland has been appointed Dental Surgeon and Mr. F. Coleman Assistant Dental Surgeon. Mr. R. C. Elmslie has been appointed Demonstrator of Pathology and Dr. H. Pritchard and Mr. H. G. Ball have been elected Junior Demonstrators of Pathology. Dr. H. G. Adamson has been appointed chief assistant in the Department for Diseases of the Skin.

Dr. C. M. H. Howell has been elected Junior Demonstrator of Physiology, and Mr. T. S. Lukis and Mr. C. T. Neve have been appointed Assistant Demonstrators of Biology.

The following awards of scholarships and prizes have been made during the year 1906-1907 :—Lawrence Scholarship, G. T. Burke, J. C. Mead (equal); Brackenbury Medical Scholarship, E. A. Cockayne; Brackenbury Surgical Scholarship, P. L. Giuseppi; Matthews Duncan Prize, R. B. S. Sewell; Kirkes Scholarship and Gold Medal, G. T. Burke; Walsham Prize, P. L. Giuseppi; Bentley Prize, H. J. Cates; Hichens Prize, S. Dixon; Wix Prize, A. W. J. Cunningham; Senior Scholarship, A. P. Fry; Junior Scholarship, R. G. Hill, C. D. Kerr, J. W. Trevan (equal); Sir George Burrows Prize, A. W. G. Woodforde; Skynner Prize, P. L. Giuseppi; Harvey Prize, K. Bremer; Treasurer's Prize, R. G. Hill; Foster Prize, W. C. Dale; Shuter Scholarship, R. R. Armstrong.

